# Opposite alterations of 5­HT_2A_ receptor brain density in subjects with schizophrenia: relevance of radiotracers pharmacological profile

**DOI:** 10.1038/s41398-021-01430-7

**Published:** 2021-05-20

**Authors:** Rebeca Diez-Alarcia, Carolina Muguruza, Guadalupe Rivero, Aintzane García-Bea, Vanessa Gómez-Vallejo, Luis F. Callado, Jordi Llop, Abraham Martín, J. Javier Meana

**Affiliations:** 1 Department of Pharmacology, University of the Basque Country, Leioa, Bizkaia Spain; 2 Centro de Investigación Biomédica en Red de Salud Mental (CIBERSAM), Leioa, Bizkaia, Spain; 3 Biocruces Bizkaia Health Research Institute, Barakaldo, Bizkaia Spain; 4 CIC BiomaGUNE, Basque Research and Technology Alliance (BRTA), Donostia/San Sebastian, Spain; 5 Centro de Investigación Biomédica en Red de Enfermedades Respiratorias (CIBERES), Madrid, Spain; 6 Achucarro Basque Center for Neuroscience, Leioa, Bizkaia Spain; 7 IKERBASQUE Basque Foundation for Science, Bilbao, Spain

**Keywords:** Diagnostic markers, Molecular neuroscience

## Abstract

The status of serotonin 5­HT_2A_ receptors (5­HT_2A_Rs) in schizophrenia has been controversial. In vivo positron emission tomography neuroimaging and in vitro post-mortem binding studies have reported conflicting results about 5­HT_2A_R density. Radiotracers bind different receptor conformations depending on their agonist, antagonist or inverse agonist properties. This study investigates 5­HT_2A_R density in the post-mortem prefrontal cortex from subjects with schizophrenia and controls using three radiotracers with a different pharmacological profile. The specific binding parameters of the inverse agonist [^18^F]altanserin, the agonist [^3^H]lysergic acid diethylamide (LSD) and the antagonist [^3^H]MDL100907 to brain cortex membranes from 20 subjects with schizophrenia and 20 individually matched controls were evaluated under similar methodological conditions. Ten schizophrenia subjects were antipsychotic-free at death. Saturation curve analyses were performed by non-linear regression to obtain a maximal density of binding sites (*B*_max_) and the affinity of the respective radiotracers (*K*_d_). In schizophrenia subjects, 5-HT_2A_R density was decreased when quantified by [^18^F]altanserin binding, whereas increased when evaluated by [^3^H]LSD binding. However, [^3^H]MDL100907 binding was unaltered. A slight loss of affinity (higher *K*_d_) was observed exclusively in [^3^H]LSD binding. The findings were more evident in antipsychotic-free subjects than in antipsychotic-treated subjects. In conclusion, a higher proportion of the 5-HT_2A_R-active functional conformation, which is rather identified by agonist radiotracers, was observed in schizophrenia patients. A consequent reduction of the inactive 5-HT_2A_R conformation, which is preferentially identified by inverse agonist radiotracers, was also obtained. Antagonist radiotracers do not distinguish between molecular conformations of the receptor, and accordingly, the absence of changes was shown. These results are compatible with the proposed increased functional activity of brain cortical 5-HT_2A_Rs in schizophrenia.

## Introduction

A role for serotonin (5-HT) in the pathophysiology and therapeutics of schizophrenia is supported by converging observations^1^. First, similarities between psychotic states in psychiatric disorders and the effects of lysergic acid diethylamide (LSD) and other 5-HT receptor-mediated psychedelic drugs (e.g., mescaline and psilocybin) have been described^2^. Second, cortical serotonin 5­HT_2A_ receptors (5-HT_2A_Rs) seem to be the critical target to induce these psychosis-like responses^3^, and second-generation antipsychotics such as clozapine, risperidone and olanzapine, among others, display potent antagonism properties on 5-HT_2A_Rs. Furthermore, since 5-HT plays a key role in emotional processing, it has been proposed that dysregulation of 5-HT neurotransmission could underlie the negative symptoms of schizophrenia^1^. The 5-HT receptor brain density is typically assessed in vivo using positron emission tomography (PET)^4^ and in vitro using post-mortem tissue homogenates and sections (for a review, see Supplementary Table S1). Evidence shows a lower density of 5-HT_2A_Rs in the frontal cortex of antipsychotic-­naive schizophrenic patients when evaluated with the very selective (200- to 500-fold higher affinity for 5-HT_2A_Rs vs. dopamine D_2_ receptors (D_2_Rs)) radiotracer [^18^F]altanserin^5–7^. In contrast, inconclusive results were obtained with other radiotracers such as [^18^F]setoperone and [^18^F]N-methyl spiperone^8–11^, which have 10- to 25-fold 5-HT_2A_R selectivity vs. dopamine D_2_Rs^7^. Since D_2_Rs are clearly involved in schizophrenia pathophysiology and treatment, the use of these radiotracers with substantial D_2_R affinity is considered a source of bias for 5-HT_2A_R detection. On the other hand, important differences among studies have also been obtained from in vitro post-mortem studies in the brain of subjects with schizophrenia^1,3,12^. Thus, while some studies described up-regulation of 5­HT_2A_Rs, others pointed towards the absence of alterations or even a down-regulation in the number of binding sites. These apparent discrepancies among post-mortem studies have been considered in the context of different demographic and clinical parameters, the existence of diverse pharmacological treatments and the variety of methodological approaches^3,12–14^. Moreover, as drug-free populations are difficult to obtain for post-mortem studies, the existence of long-term antipsychotic treatment has been considered the main explanatory factor for differences between in vivo neuroimaging studies in drug-naive patients and in vitro findings in post-mortem brain. However, less attention has been paid to the pharmacological properties, such as agonist, antagonist or inverse agonist, of the respective drugs used as radiotracer tools to identify 5­HT_2A_Rs. The most common 5­HT_2A_R drugs used to generate radioligands for in vivo PET studies ([^18^F]altanserin and [^11^C]M100907) are considered antagonists, and the efforts towards the development of agonist radiotracers have reported limited success^15^. In marked contrast, post-mortem studies of 5­HT_2A_R quantitation in schizophrenia have been performed with the agonist [^3^H]LSD and the partial agonist [^3^H]ketanserin radiotracers (Supplementary Table S1). Although unattended, the pharmacological properties, such as agonist, antagonist or inverse agonist, are determinants for the receptor conformation identified by the radiotracer and, subsequently, for the estimated binding density^16^.

**Fig. 1:**
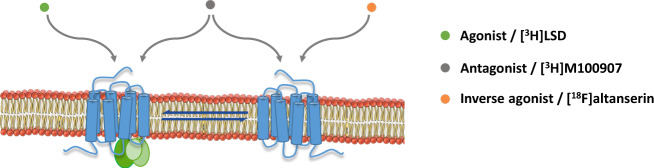
Conformational states of the 5-HT_2A_R. The receptor conformation coupled to G-protein signalling pathways represents the functional state and is preferentially identified by agonist radioligands such as [^3^H]LSD. The inactive conformation of the 5-HT_2A_R, which is uncoupled from G proteins, is preferentially labelled by inverse agonist radioligands such as [^18^F]altanserin. Active and inactive conformations are interchangeable states that stay in equilibrium^17^. Antagonist radioligands, such as [^3^H]MDL100907, bind both receptor conformations and, therefore, do not distinguish between functional and non-functional states.

**Fig. 2:**
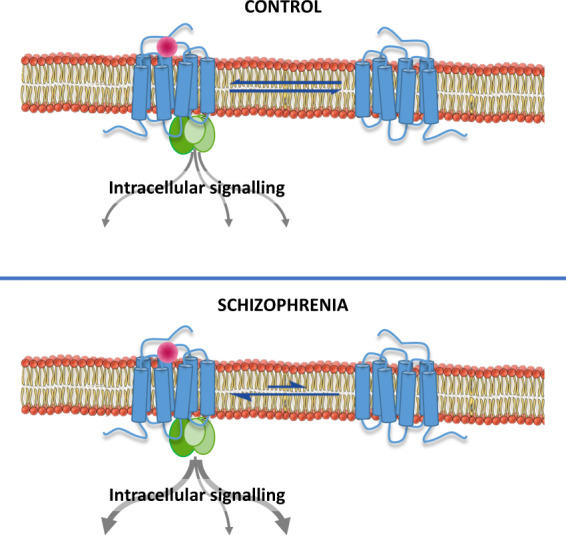
Representation of the imbalance between 5-HT_2A_R conformations towards the state preferentially labelled by agonists observed in the prefrontal cortex of subjects with schizophrenia. The disequilibrium promotes a G-protein overactivation in response to 5-HT_2A_R agonists^20^. Based on this hypothesis, agonist radioligands should identify higher binding sites and inverse agonist radioligands should label lower binding sites in schizophrenia than in controls. Antagonist radioligands would not discriminate between schizophrenia and control subjects.

G-protein-coupled receptors (GPCRs) and, among them, 5­HT_2A_Rs display different molecular conformations that are interchangeable and stay in equilibrium^17^. Thus, the receptor conformation that couples to G proteins is considered to be functionally active and represents the high-affinity state of the receptor, which is preferentially identified by agonist radioligands. Conversely, inverse agonist radioligands show preference to bind the low-affinity state, i.e., the inactive receptor conformation, which is uncoupled from G proteins. Finally, antagonist radioligands bind with similar affinity to both receptor conformations (Fig. 1). Therefore, the binding of agonist radioligands to the G-protein-coupled conformation should serve as a more accurate measure of 5­HT_2A_R functions and dysfunctions than antagonist binding^18,19^. In vitro studies have revealed increased functional coupling of 5­HT_2A_Rs to G proteins in the brain cortex of subjects with schizophrenia without alterations in total values of receptor density^20^. This finding suggests that an imbalance of 5­HT_2A_Rs towards the high-affinity receptor conformation might be present in schizophrenia, leading to overactive G-protein-dependent signalling. Under this 5­HT_2A_R overactivity, enhanced agonist radioligand binding should be expected. Conversely, a decreased binding of inverse agonist radioligands to the uncoupled conformation might indicate a decrease of the low-affinity receptor state and prove the existence of an imbalance between coupled and uncoupled 5­HT_2A_R conformations in schizophrenia, with receptor equilibrium displaced towards the active conformational state (Fig. 2).

Although [^18^F]altanserin and [^11^C]MDL100907 were initially developed as selective antagonist radiotracers to quantify 5­HT_2A_Rs in PET studies^15^, altanserin has recently been demonstrated to show inverse agonist properties on 5­HT_2A_Rs in post-mortem human brain^13,21^. In fact, [^18^F]altanserin binding is decreased in the brain of patients with schizophrenia^5,6^ and in prodromal at-risk state subjects^22^. In contrast to PET evaluations, studies in post-mortem brain of subjects with schizophrenia have mainly been performed with the agonist [^3^H]LSD and the partial agonist [^3^H]ketanserin radiotracers (Supplementary Table S1). Noticeably, among post-mortem studies, those developed in antipsychotic-free schizophrenia cases displayed enhanced 5­HT_2A_R density in the prefrontal cortex^13,23,24^. Based on all these observations, it could be hypothesized that 5­HT_2A_R agonists and inverse agonists identify different molecular conformations of this receptor in schizophrenia, leading to opposite findings. However, the 5­HT_2A_R receptor binding of agonist, antagonist and inverse agonist radiotracers has never been tested under the same methodological conditions.

The aim of the present study was to investigate the 5­HT_2A_R density in post-mortem prefrontal cortex of subjects with schizophrenia, using three different radiotracers with different intrinsic activity properties (agonist, antagonist and inverse agonist) on this receptor. [^3^H]LSD, [^3^H]MDL100907 and [^18^F]altanserin were selected as representative radiotracers with well-established pharmacological profiles. LSD is a hallucinogenic drug with 5­HT_2A_R partial agonist properties^25,26^, and its ^3^H-labelled form identifies this receptor under blocking conditions for other 5-HT receptors. MDL100907 is a very selective 5­HT_2A_R antagonist^27^, whose ^18^F- and ^11^C-labelled forms have been used for PET studies^15,28^. Altanserin is a highly selective 5­HT_2A_R inverse agonist^21^, commonly considered as antagonist PET radiotracer when labelled with ^18^F^15^. The three radiotracers were evaluated on saturation binding experiments in brain cortex membrane homogenates of the same subjects and under similar experimental conditions. We sought to test the hypothesis that different alterations of 5­HT_2A_R density are obtained in schizophrenia depending on the intrinsic activity properties of each radiotracer. The results would shed light on the unclarified status of brain 5­HT_2A_Rs in schizophrenia.

## Subjects, materials and methods

### Post-mortem human brain samples

Brain samples were obtained at autopsies performed in the Basque Institute of Legal Medicine, Bilbao, Spain, in compliance with policies of research and ethical boards for post-mortem brain studies. Deaths were subjected to retrospective searching for previous medical diagnosis and treatment using the examiner’s information and records from hospitals and mental health centres. A total number of 20 brains from subjects with ante-mortem diagnosis of schizophrenia according to the Diagnostic and Statistical Manual of Mental Disorders (DSM-IV, DSM-IV-TR) were matched to 20 brains from control subjects in a paired design. Control subjects were chosen among the collected brains on the basis of the absence of neuropsychiatric disorders or drug abuse, and an appropriate sex, storage time and post-mortem interval (elapsed time between death and tissue dissection/freezing) to match each schizophrenia subject. Toxicological screening of blood samples of all subjects was performed to determine the presence at death of antipsychotics, other drugs and ethanol. According to the absence or presence of antipsychotic drugs in this toxicological screening, schizophrenia population was divided into two groups, a group of antipsychotic-free (*n* = 10) and a group of antipsychotic-treated (*n* = 10) subjects. Schizophrenia and control groups were similar for sex ratio, age, storage time and post-mortem interval values (Supplementary Table S2). Seventeen out of the 20 subjects in the schizophrenia group committed suicide. Matched control subjects mainly died by accidental causes. Therefore, the mechanism of death indicates that almost all were violent or sudden. Specimens of the dorsolateral prefrontal cortex (Brodmann’s area 9) were dissected at autopsy following standard procedures and immediately stored at −80 °C until assayed. A complete description of the whole population of subjects with schizophrenia and their individually matched controls can be found in Supplementary Table S2. Some of the schizophrenia cases and controls have been previously used to evaluate the [^3^H]ketanserin binding density^13,24^ and G-protein activation mediated by 5-HT_2A_Rs^20,29^ (see Supplementary Table S2 for details). The prefrontal cortex was selected as the region of interest based on the morphological alterations associated to schizophrenia^30^ and the large expression of 5-HT_2A_Rs in the area^5^.

### Materials and drugs

MDL100907 (volinanserin) and altanserin were purchased from Sigma-Aldrich. [^3^H]LSD (86.3 Ci/mmol at delivery time) was obtained from PerkinElmer Life and Analytical Sciences, Inc., and [^3^H]MDL100907 (80 Ci/mmol at delivery time) was obtained from ARC Radiochemicals. All other chemicals were obtained from standard sources.

### Synthesis of [^18^F]altanserin

[^18^F]altanserin was produced in a TRACERlab FXFN synthesis module (GE Healthcare) by radiofluorination of nitro-altanserin (ABX, Radeberg, Germany) as previously published^31^. Specific activity at initial incubation time (see below) was in the range 300–700 GBq/μmol. The average radiochemical yield was 11 ± 4% (end of synthesis). Radiochemical purity was >97% in all cases.

### Brain membranes preparation

Brain cortex samples were processed to obtain membrane-enriched homogenates as previously described^13^.

### [^18^F]Altanserin, [^3^H]LSD and [^3^H]MDL100907 binding assays

Complete saturation binding assays were performed with [^18^F]altanserin (0.03–4 nM, eight concentrations), [^3^H]LSD (0.03–10 nM, ten concentrations) and [^3^H]MDL100907 (0.007–4 nM, ten concentrations) in order to determine the density (*B*_max_) and the affinity (*K*_d_) of 5­HT_2A_Rs. Incubation was carried out in tubes ([^18^F]altanserin) or 96-well plates ([^3^H]LSD and [^3^H]MDL100907) and started with the addition of the brain membrane preparation. Reactions were incubated for 40 min at 37 °C for [^18^F]altanserin binding assays and 90 min at 37 °C for [^3^H]LSD and [^3^H]MDL100907 binding assays. The presence of MDL100907 (1 µM) or altanserin (10 µM) was used to determine the non-specific binding of [^18^F]altanserin and [^3^H]LSD, and of [^3^H]MDL100907, respectively. After incubation, free radioligand was separated from bound radioligand by rapid filtration under vacuum through GF/C glass fibre filters pre-soaked with 0.5% polyethylenimine and counted for radioactivity gamma counting ([^18^F]altanserin; 2470 WIZARD2 Automatic Gamma Counter, PerkinElmer) or by liquid scintillation ([^3^H]LSD and [^3^H]MDL100907; MicroBeta TriLux Counter, PerkinElmer). Results were corrected for each radiotracer decay. Pairs of cases and controls were always processed at the same time and all samples were run in duplicate.

### Data and statistical analyses

Data obtained from saturation binding experiments of each subject were analysed by non-linear regression using the GraphPad Prism™ software. The apparent equilibrium dissociation constant (*K*_d_) and the maximum density of specific binding sites (*B*_max_) were obtained. *K*_d_ values were normalized to log transformation before parametric analyses. All data were subjected to a Grubbs’s test in order to detect and reject possible outlier values among experimental groups. Pearson’s correlation *r* coefficient was calculated to test for a possible association between independent covariables (age, post-mortem interval and storage time) and receptor density. When correlation was significant, analysis of covariance was performed controlling for the independent covariable. One-way analysis of variance followed by Bonferroni’s post-hoc test was used to compare results between radioligands. Results are expressed as mean±standard deviation (SD) of individual values.

Statistical comparisons between groups were conducted by non-linear curve-fitting co­analysis of all individual binding experiments. The selection between a single-curve model (absence of differences between groups) and a two-curve model (statistical differences between groups) was made by the extrasum-of-squares *F* test using GraphPad Prism™. When statistical differences between curves were obtained, further individual contrasts were performed to detect whether differences were attributable to changes in *B*_max_ and/or *K*_d_ values between groups^32,33^. The analysis that permitted one or more of the parameters to be shared without a significant increase in the residual variance was taken as the best fit. In this non-linear analysis, results (*B*_max_ and *K*_d_) are expressed as the best estimation parameter ± SD. These values obtained from simultaneous non-linear regression analyses were not used for parametric statistical calculations.

In all analyses, the level of statistical two-tailed significance was chosen as *p* = 0.05.

## Results

### Specific binding sites for [^18^F]altanserin, [^3^H]LSD and [^3^H]MDL100907

The individual non-linear analysis of each radioligand binding fitted to a saturation curve displaying a single specific binding site of high affinity, compatible with selective detection of 5-HT_2A_Rs. The receptor density (*B*_max_) in the overall population was similar when identified by [^18^F]altanserin or [^3^H]MDL100907 binding, while it was significantly higher when estimated by [^3^H]LSD binding (192 ± 82% over [^18^F]altanserin, *p* < 0.01; 210 ± 89% over [^3^H]MDL100907, *p* < 0.0001). The binding affinities, expressed by *K*_d_ values, were always in the nanomolar range (*K*_d_ = 0.36 ± 0.19 nM for [^18^F]altanserin; *K*_d_ = 1.26 ± 0.86 nM for [^3^H]LSD; *K*_d_ = 0.47 ± 0.37 nM for [^3^H]MDL100907) without significant differences between radioligands.

Positive correlations between densities obtained with [^18^F]altanserin and [^3^H]MDL100907 (*r* = 0.332, *p* < 0.05), and between [^3^H]MDL100907 and [^3^H]LSD (*r* = 0.894, *p* < 0.0001) were found. In contrast, no significant correlation was found between densities obtained with [^18^F]altanserin and [^3^H]LSD, suggesting that these radioligands could identify different binding populations.

### Effects of demographic parameters and post-mortem conditions

The density of [^18^F]altanserin binding sites displayed a negative correlation with the age at death (*r* = −0.341; *p* < 0.05). According to this linear decay, the average decrease per decade for 5-HT_2A_Rs was 35 ± 15 fmol/mg. In the case of [^3^H]MDL100907 binding, there was also a decrease of density with age (30 ± 17 fmol/mg per decade) that did not reach significant correlation (*r* = −0.283; *p* = 0.08). No correlation between age at death and density of [^3^H]LSD binding sites was obtained.

[^18^F]altanserin, [^3^H]LSD and [^3^H]MDL100907 binding properties were not significantly affected neither by post-mortem interval nor by storage time at −80 °C.

### Comparison between schizophrenia and control groups

**Fig. 3:**
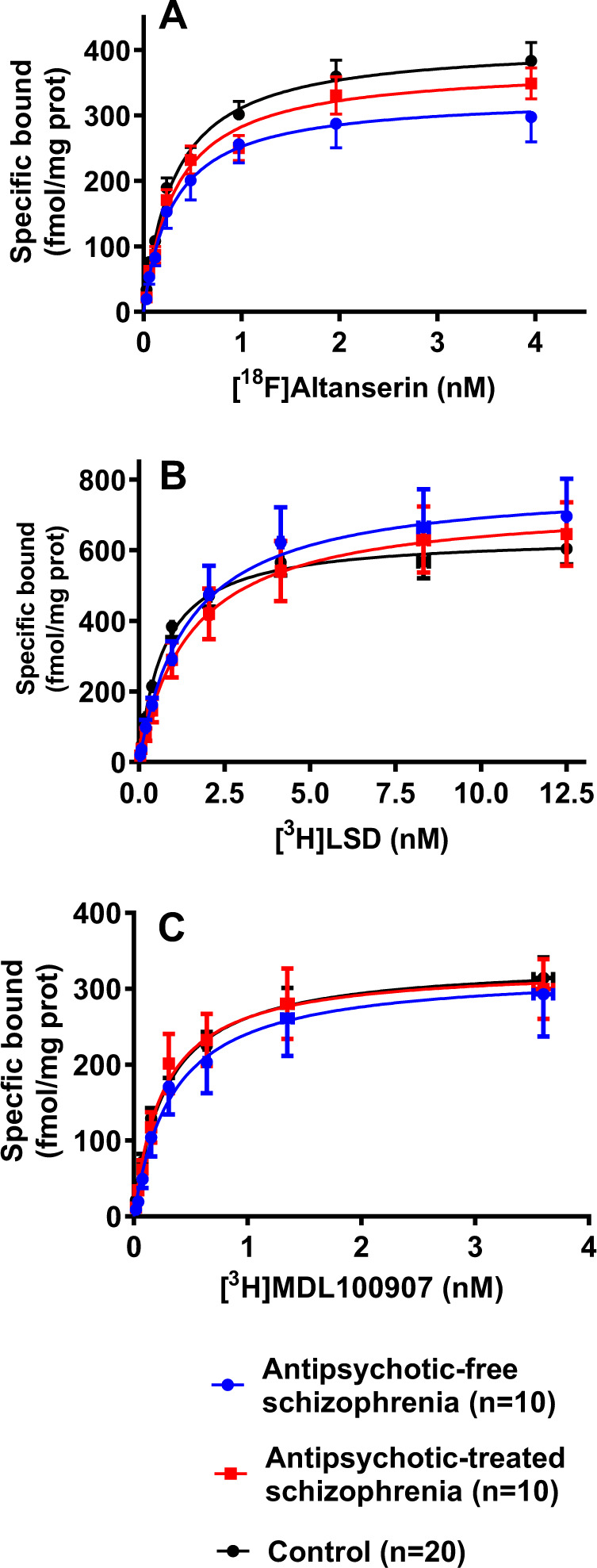
Alterations of 5-HT_2A_ receptor density in schizophrenia. Saturation curves of the specific [^18^F]altanserin (**A**), [^3^H]LSD (**B**) and [^3^H]MDL100907 (**C**) binding to membranes of post-mortem frontal cortex from subjects with schizophrenia and presence of antipsychotic drugs in the blood at the time of death (antipsychotic-treated), subjects with schizophrenia and no antipsychotic drugs in the blood (antipsychotic-free), and matched control subjects. Each schizophrenia group was analysed vs. the respective control group, but in order to clarify, here the two control groups are shown together. The curves represent the best-fit estimation generated by the non-linear co-analysis of individual results in each group. The density of 5-HT_2A_Rs is expressed as the asymptote value (*B*_max_) of the radioligand bound. The affinity of the radioligand is expressed by the concentration of radioligand that promotes the half-maximal bound (*K*_d_). Points representing means were not used for statistical analysis.

The co-analysis of saturation curves obtained with [^18^F]altanserin showed a statistically significant reduction of the density of the binding sites in the schizophrenia group compared to matched controls. No differences in affinity were detected between schizophrenia and control groups (Table 1). When the presence of antipsychotic drugs was considered, the co-analysis demonstrated a significant reduction of [^18^F]altanserin binding sites in antipsychotic-free subjects with schizophrenia (*B*_max_ = 329 ± 24 fmol/mg) vs. matched controls (*B*_max_ = 410 ± 25 fmol/mg) (*p* < 0.05) (Fig. 3A). In contrast, subjects with schizophrenia and presence of antipsychotic treatment displayed density values (*B*_max_ = 376 ± 18 fmol/mg) closer to the respective matched control group (*B*_max_ = 411 ± 23 fmol/mg) (Fig. 3A). No changes were observed in the affinity parameters (*K*_d_ = 0.30 ± 0.07 nM in antipsychotic-free schizophrenia group vs. *K*_d_ = 0.34 ± 0.07 nM in matched controls; *K*_d_ = 0.33 ± 0.05 nM in antipsychotic-treated schizophrenia group vs. *K*_d_ = 0.29 ± 0.06 nM in matched controls). As expected, these findings were confirmed by analysis of covariance controlling *B*_max_ value for age.

**Table 1:** Radioligand binding parameters in the prefrontal cortex of subjects with schizophrenia and matched controls.

	Schizophrenia			Control			*F*[d.f.,d.f.]	*p* Value
	*B*_max_ (fmol/mg)	*K*_d_ (nM)	*n*	*B*_max_ (fmol/mg)	*K*_d_ (nM)	*n*		
	Mean ± SD	Mean ± SD		Mean ± SD	Mean ± SD			
[^18^F]Altanserin	352 ± 15	0.32 ± 0.05	20	410 ± 17	0.32 ± 0.05	20	*F*[1,288] = 6.361	0.0122
[^3^H]LSD	765 ± 47	1.5 ± 0.32	20	640 ± 23	0.73 ± 0.11	20	F[1,311] = 8.282	0.0043
[^3^H]MDL100907	324 ± 23	0.29 ± 0.07	20	335 ± 16	0.28 ± 0.04	20	*F*[1,294] = 0.354	0.7023

For each radioligand, the two sets of data (schizophrenia and control) were first analysed separately. The overall value for the sum of squares and the degrees of freedom (d.f.) was the sum of the individual values of each fit. Next, the two sets of data were pooled and analysed simultaneously constraining them to share one or two common parameters (*B*_max_, *K*_d_). The pooled fit yielded values for the sum of squares and degrees of freedom. The analysis that permitted one or two parameters to be shared without a significant increase in the residual variance was taken as the best fit. [^18^F]Altanserin and [^3^H]LSD binding curves were considered different between schizophrenia and control. The subsequent analysis demonstrated that statistical differences were adscribed to different *B*_max_ but not to *K*_d_ values. The *F*, d.f. and *p* values displayed correspond to this condition. For [^3^H]MDL100907, estimations obtained under equivalent analysis are shown.

The co-analysis of saturation curves obtained with [^3^H]LSD demonstrated a significant increase of binding sites in subjects with schizophrenia compared to matched controls The co-analysis also demonstrated higher *K*_d_ values of this radioligand in the schizophrenia group than in controls (Table 1). When the presence of antipsychotic drugs in blood was considered, the enhanced receptor density was maintained in antipsychotic-free schizophrenia subjects (*B*_max_ = 791 ± 69 fmol/mg) when compared with matched controls (*B*_max_ = 646 ± 34 fmol/mg) (*p* < 0.05) (Fig. 3B). Conversely, antipsychotic-treated schizophrenia subjects displayed receptor density values (*B*_max_ = 735 ± 63 fmol/mg) that did not differ from those in respective control group (*B*_max_ = 635 ± 30 fmol/mg) (Fig. 3B). In the case of K_d_ values, the increase was significant for both antipsychotic-free (K_d_ = 1.45 ± 0.45 nM) and antipsychotic-treated (*K*_d_ = 1.52 ± 0.44 nM) schizophrenia subjects compared with respective controls (*K*_d_ = 0.69 ± 0.15 nM and *K*_d_ = 0.77 ± 0.15 nM) (*p* < 0.05). Re-evaluation with age as covariate maintained similar results. In order to test whether the residual presence of antipsychotic drugs could contribute to the increased *K*_d_ of [^3^H]LSD binding, a correlation between published *K*_i_ for 5-HT2AR values^34,35^ of drugs detected in the post-mortem toxicological screening and individual *K*_d_ values obtained for [^3^H]LSD was performed. A significant correlation (*r* = 0.68, *p* = 0.04) was obtained in antipsychotic-treated subjects.

The co-analysis of curves obtained with [^3^H]MDL100907 showed no differences of binding sites between subjects with schizophrenia and matched controls. The affinities were also similar (Table 1). No differences were detected neither in antipsychotic-free schizophrenia subjects (*B*_max_ = 324 ± 37 fmol/mg; *K*_d_ = 0.34 ± 0.13 nM) with respective controls (*B*_max_ = 328 ± 23 fmol/mg; *K*_d_ = 0.32 ± 0.07 nM) nor in antipsychotic-treated subjects (*B*_max_ = 330 ± 28 fmol/mg; *K*_d_ = 0.26 ± 0.07 nM) vs. matched controls (*B*_max_ = 344 ± 22 fmol/mg; *K*_d_ = 0.25 ± 0.05 nM) (Fig. 3C). Age at death did not influence the results in the analysis of covariance.

## Discussion

The present study demonstrates in post-mortem human frontal cortex that alterations of 5-HT_2A_Rs observed in schizophrenia are dependent on the pharmacological properties of the radiotracer used to identify this receptor. Thus, binding assays with an agonist ([^3^H]LSD), an antagonist ([^3^H]MDL100907) and an inverse agonist ([^18^F]altanserin) radiotracer conducted in similar incubation conditions from identical samples lead to different results. The present study provides evidence that the agonist radioligand binding to 5-HT_2A_Rs is increased in schizophrenia, whereas inverse agonist radioligand binding is reduced, and the antagonist radioligand binding remains unaltered. This differential pattern is remarkable in antipsychotic-free subjects, whereas the presence of these drugs in blood tends to reverse the 5-HT_2A_R density alterations to control values. Until recently, an equivalent PET study was not feasible, mainly due to the lack of suitable agonist radiotracers for selective 5-HT_2A_R identification^36^. However, the recent development of [^11^C]Cimbi-36^15,18^, a non-selective 5-HT_2A/B/C_R agonist^37^, could help to confirm through head-to-head in vivo comparisons between antagonist and inverse agonist radiotracers the findings here reported in the post-mortem tissue. Nevertheless, the technical and ethical feasibility of in vivo identification of 5-HT_2A_Rs by three different PET radiotracers in the same patient and short time frame is limited. Therefore, in vitro post-mortem studies could help to overcome these drawbacks in the study of neurotransmitter receptor molecular alterations. However, other potentially confounding factors, especially the existence of previous treatment with antipsychotic drugs, add limitations to the conclusions of post mortem studies. In fact, the number of antipsychotic-free subjects included in this type of studies is very limited^38^ (Supplementary Table S1).

The increased density of 5-HT_2A_Rs identified by the agonist radiotracer [^3^H]LSD confirms the proposed higher functional sensitivity of this receptor in schizophrenia. Previous studies with the partial agonist radioligand [^3^H]ketanserin have reported similar findings in antipsychotic-free subjects^13,24^. Endogenous and exogenous agonists bind preferentially to the high-affinity state of the receptor, which represents the active functional conformation coupled to cell signalling pathways (Fig. 1). Recently, the assessment of 5-HT_2A_R coupling to G proteins in post-mortem frontal cortex has demonstrated an enhanced sensitivity of inhibitory G_i1_ proteins in response to the agonist (±)DOI (2,5-dimethoxy-4-iodoamphetamine) in schizophrenia^20^. In concordance, the prolactin response to the 5-HT-releasing drug d-fenfluramine^39^, which is considered a functional in vivo test dependent on 5-HT_2A/C_Rs activation, is enhanced in drug-free schizophrenia subjects^40,41^. Therefore, evidence points towards a functional hyperactivity of 5-HT_2A_Rs in schizophrenia. This issue has resulted in controversy since PET studies with [^18^F]altanserin have suggested decreased binding potential in antipsychotic-naive schizophrenia patients^5,6^. The present study illustrates how the reduction of [^18^F]altanserin binding (−20%) is compatible with enhanced binding (+22%) of agonist radiotracers as [^3^H]ketanserin and [^3^H]LSD. The compound altanserin has been classically regarded as the selective 5-HT_2A_R antagonist. However, in the brain cortex, this drug shows inverse agonist properties^13,21^, which means preferential labelling to the receptor conformation uncoupled from G proteins. In this way, the pharmacological profile of [^18^F]altanserin explains the reduced 5-HT_2A_R density reported in schizophrenia as a reduction of the uncoupled conformation of this receptor. G-protein-coupled (higher affinity for agonists than for inverse agonists) and G-protein-uncoupled (higher affinity for inverse agonists than for agonists) receptor conformations are interchangeable molecular states of GPCRs. In brains of subjects with schizophrenia, the imbalance between 5-HT_2A_R conformations in favour of the G-protein-coupled state would be expressed as increased density of the agonist [^3^H]LSD binding and reduced density of the inverse agonist [^18^F]altanserin binding, as observed in the present study (Fig. 2). Moreover, this hypothesis should be supported by the concurrent absence of changes in the 5-HT_2A_R density delineated by selective antagonist radiotracers. Neutral antagonist drugs do not distinguish among molecular conformations of the receptor and, thereby, are not suitable tools to detect the existence of imbalance between 5-HT_2A_R conformational states in schizophrenia. The absence of differences for the antagonist [^3^H]MDL100907 binding between schizophrenia and control groups obtained in the present study agrees with this argument and supports the existence of a functional 5-HT_2A_R imbalance in the pathophysiology of the disorder. More recent post-mortem studies have added further weight to this hypothesis by showing that messenger RNA expression and total protein immunodetection of 5-HT_2A_Rs are unaltered in subjects with schizophrenia free of antipsychotic treatment^20^.

**Fig. 4:**
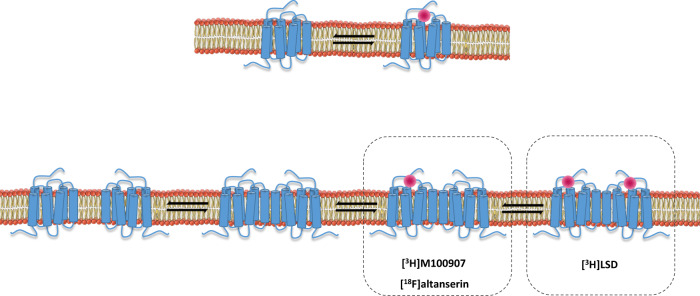
Monomer and oligomer conformations of the 5-HT_2A_R. The 5-HT_2A_R is expressed as single monomers but also as receptor complexes constituted by two or more receptor units. The binding of a drug to one of the monomers of the complex could induce positive, negative or no cooperativity for the binding to other monomers with the complex. For example, the binding of the antagonist MDL100907 or the inverse agonist altanserin to one of the binding pockets of the homodimer introduces negative allosteric effects on the binding of the same drug to the second binding pocket^43^. This negative cooperativity would prevent the identification of the total number of binding sites. In contrast, the binding of hallucinogenic 5-HT_2A_R agonist such as (±)DOB and LSD to receptor homodimers does not modify the estimation of the total number of binding sites^43^. Higher order 5-HT_2A_R oligomeric and heteromeric complexes of 5-HT_2A_Rs with other GPCRs are also feasible^24,42^.

One apparent inconsistency of the present study is the different receptor binding density obtained between radiotracers. [^3^H]LSD approximately identified a two-fold higher number of binding sites than [^18^F]altanserin and [^3^H]MDL100907. It is widely accepted that 5-HT_2A_Rs are assembled into homodimeric and heterodimeric structures^24,42^. Receptor oligomers coexist with monomeric forms (Fig. 4). Dimeric receptor complex crosstalk to each other promoting differential modulation of the ligand access to the respective binding pockets^36^. In fact, the binding of partial agonist and antagonists, as ketanserin and MDL100907, to one of the two binding sites in the 5-HT_2A_R homodimer introduces negative cooperativity effects on the propensity of a second molecule of the same drug to bind the dimer^43^. In contrast, the hallucinogenic 5-HT_2A_R agonist (±)DOB shows a similar affinity for the two binding sites of the dimer^43^. Therefore, it is feasible to propose that [^3^H]LSD is able to label the two binding sites of the homodimeric 5-HT_2A_R, whereas [^3^H]MDL100907, [^18^F]altanserin and [^3^H]ketanserin binding to one of the receptor pockets prevent the own radioligand binding to the second site (Fig. 4). This molecular mechanism would be reflected in a two-fold higher density when estimated by radiotracers bound to the full homodimer with respect to the density obtained by radiotracers bound only to one of the monomers that conform to the dimer. The results shown in the present study together with those in previous studies with the radiotracer [^3^H]ketanserin^13,24^ agree with this hypothesis of a homodimeric 5-HT_2A_R structure and function. Certainly, the assumption of the receptor oligomerization paradigm should affect future comparisons between radiotracer binding properties.

The observed decline of 5-HT_2A_R density with ageing is a repeated finding in previous post-mortem^13,24,44^ and PET^45^ studies. This profound effect of ageing provides the rationale for experimental designs based on one-to-one individual matching of each schizophrenia case with respective control, as performed here, rather than the usual and less rigorous group-based matching.

The presence or absence at death of antipsychotic drugs in the blood of subjects with schizophrenia represents another relevant confounding factor in radioligand binding studies (Supplementary Table S1). In the present study, the absence of antipsychotic drugs in the toxicological analysis does not mean that these subjects termed as antipsychotic-free were antipsychotic-naive, but rather that they were untreated in the nearest ante-mortem period. The more [^18^F]altanserin binding and the less [^3^H]LSD binding densities in antipsychotic-treated respect to antipsychotic-free schizophrenia subjects suggest that antipsychotic treatment would counterbalance the 5-HT_2A_Rs alterations observed in schizophrenia. Long-term treatment with second-generation antipsychotics modulates 5-HT_2A_R expression in animals^14,24^ and could modify binding parameters due to residual presence of antipsychotics acting as 5-HT_2A_R antagonists^20^. However, the possibility that observed alterations of 5-HT_2A_R density (*B*_max_) in schizophrenia represent a consequence of the current or past long-term antipsychotic treatment is improbable. First, the changes of density are more evident in recent antipsychotic-free than in antipsychotic-treated subjects. Second, the differential up- or down-regulation of 5-HT_2A_Rs associated with recent antipsychotic treatment in function of the different radiotracers makes unlikely a residual competitive effect between the antipsychotic and the radioligand to bind the receptor pocket. In contrast to density (*B*_max_), the apparent affinity (*K*_d_ value) was sensitive to the residual presence of antipsychotic drugs, although this effect was only observed for [^3^H]LSD binding assays. The finding reasserts the use of agonist radiotracers to better detect the 5-HT_2A_R occupation by psychedelic drugs^46^. Receptor exploration in drug-free conditions is more feasible by PET imaging than by retrospective post-mortem binding studies. Indeed, most of the post-mortem studies evaluating 5-HT_2A_Rs in schizophrenia have been performed in the brain of subjects under antipsychotic treatment, which probably led to inconclusive results (Supplementary Table S1). Besides this, when schizophrenia subjects were differentiated between those under antipsychotic treatment and those antipsychotic-free, the post-mortem radioligand studies demonstrated up-regulation of brain 5-HT_2A_Rs identified by agonist/partial agonist radiotracers^13,20,23,24^. Therefore, in order to discard eventual bias in post-mortem studies, independent and well-matched groups of antipsychotic-free and antipsychotic-treated subjects should be selected and independently analysed.

Another potential confounding factor to consider in the present study is the fact that schizophrenia subjects died mostly from violent suicide mechanisms. Suicide has been proposed as a condition that could influence the evaluation of 5-HT_2A_Rs^47^. However, there are several studies in the frontal cortex of suicide victims with a variety of psychiatric disorders supporting that suicide unlikely represents a major confounder in 5-HT_2A_R binding studies^1,13,48,49^.

In conclusion, the study and interpretation of 5-HT_2A_R dysfunctions in schizophrenia requires a deep knowledge of the pharmacological properties of the candidate radiotracers. The distinction of 5-HT_2A_R radiotracers between agonist, antagonist and inverse agonist may shed light on the, up to now, contradictory results. According to the different pharmacological profile, the present results and most of the studies would demonstrate an upregulation of the active functional 5-HT_2A_R conformation in the brain of subjects with schizophrenia.

The present results support the hypothesis that 5-HT_2A_R molecular conformation and/or the receptor interaction with other synaptic proteins might be altered in schizophrenia. Moreover, as previously described, the antipsychotic treatment seems also to modify the functional state of 5-HT_2A_Rs, trying to revert the alterations found in antipsychotic-free schizophrenia subjects. Therefore, the development and in vivo use of agonist radiotracers in antipsychotic-naive patients should be encouraged to validate the 5-HT_2A_R overactivity here proposed.

## Supplementary information

Table S1

Table S2

## References

[1] Selvaraj S, Arnone D, Cappai A, Howes O (2014). Alterations in the serotonin system in schizophrenia: a systematic review and meta-analysis of postmortem and molecular imaging studies. Neurosci. Biobehav. Rev..

[2] Geyer MA, Vollenweider FX (2008). Serotonin research: contributions to understanding psychoses. Trends Pharmacol. Sci..

[3] Gonzalez-Maeso J, Sealfon SC (2009). Psychedelics and schizophrenia. Trends Neurosci..

[4] Paterson LM, Kornum BR, Nutt DJ, Pike VW, Knudsen GM (2013). 5-HT radioligands for human brain imaging with PET and SPECT. Med. Res. Rev..

[5] Rasmussen H (2010). Decreased frontal serotonin2A receptor binding in antipsychotic-naive patients with first-episode schizophrenia. Arch. Gen. Psychiatry.

[6] Rasmussen H (2016). Low frontal serotonin 2A receptor binding is a state marker for schizophrenia?. Eur. Neuropsychopharmacol..

[7] Erritzoe D (2008). Cortical and subcortical 5-HT_2A_ receptor binding in neuroleptic-naïve first-episode schizophrenia patients. Neuropsychopharmacology.

[8] Trichard C (1998). No serotonin 5-HT2A receptor density abnormality in the cortex of schizophrenic patients studied with PET. Schizophr. Res..

[9] Ngan ET, Yatham LN, Ruth TJ, Liddle PF (2000). Decreased serotonin 2A receptor densities in neuroleptic-naïve patients with schizophrenia: a PET study using [(18)F]setoperone. Am. J. Psychiatry.

[10] Verhoeff NP (2000). A voxel-by-voxel analysis of [^18^F]setoperone PET data shows no substantial serotonin 5-HT(2A) receptor changes in schizophrenia. Psychiatry Res.

[11] Okubo Y (2000). Serotonin 5-HT2 receptor in schizophrenic patients studied by positron emission tomography. Life Sci..

[12] Dean B (2003). The cortical serotonin2A receptor and the pathology of schizophrenia: a likely accomplice. J. Neurochem..

[13] Muguruza C (2013). Dysregulated 5-HT(2A) receptor binding in postmortem frontal cortex of schizophrenic subjects. Eur. Neuropsychopharmacol..

[14] Dean B, Crossland N, Boer S, Scarr E (2008). Evidence for altered post-receptor modulation of the serotonin 2a receptor in schizophrenia. Schizophr. Res..

[15] L’Estrade ET (2018). Classics in neuroimaging: the serotonergic 2A receptor system - from discovery to modern molecular imaging. ACS Chem. Neurosci..

[16] Lopez-Gimenez J (2001). Multiple conformations of native and recombinant human 5-hydroxytryptamine_2A_ receptors are labeled by agonists and discriminated by antagonists. Mol. Pharm..

[17] Battaglia G, Shannon M, Titeler M (1984). Guanyl nucleotide and divalent cation regulation of cortical S2 serotonin receptors. J. Neurochem..

[18] Ettrup A (2014). Serotonin 2A receptor agonist binding in the human brain with [^11^C]Cimbi-36. J. Cereb. Blood Flow Metab..

[19] Colom M, Vidal B, Zimmer L (2019). Is there a role for GPCR agonist radiotracers in PET neuroimaging?. Front. Mol. Neurosci..

[20] García-Bea A (2019). Serotonin 5-HT2A receptor expression and functionality in postmortem frontal cortex of subjects with schizophrenia: selective biased agonism via G_αi1_-proteins. Eur. Neuropsychopharmacol..

[21] Diez-Alarcia R (2019). Big data challenges targeting proteins in GPCR signaling pathways; combining PTML-ChEMBL models and [^35^S]GTPγS binding assays. ACS Chem. Neurosci..

[22] Hurlemann R (2008). 5-HT2A receptor density is decreased in the at-risk mental state. Psychopharmacology.

[23] Whitaker PM, Crow TJ, Ferrier IN (1981). Tritiated LSD binding in frontal cortex in schizophrenia. Arch. Gen. Psychiatry.

[24] Gonzalez-Maeso J (2008). Identification of a serotonin/glutamate receptor complex implicated in psychosis. Nature.

[25] Nichols DE (2016). Psychedelics. Pharmacol. Rev..

[26] Kim K (2020). Structure of a hallucinogen-activated Gq-coupled 5-HT_2A_ serotonin receptor. Cell.

[27] Johnson MP, Siegel BW, Carr AA (1996). [^3^H]MDL 100,907: a novel selective 5-HT2A receptor ligand. Naunyn Schmiedeberg’s Arch. Pharmacol..

[28] Talbot PS (2012). Extended characterisation of the serotonin 2A (5-HT2A) receptor-selective PET radiotracer ^11^C-MDL100907 in humans: quantitative analysis, test-retest reproducibility, and vulnerability to endogenous 5-HT tone. Neuroimage.

[29] Odagaki, Y., Kinoshita, M., Meana, J. J., Callado, L. F. & García-Sevilla, J. A. 5-HT_2A_ receptor-mediated Gα_q/11_ activation in psychiatric disorders: a postmortem study. *World J. Biol. Psychiatry*10.1080/15622975.2020.1839967 (2021). 10.1080/15622975.2020.1839967 33084439

[30] Lewis DA, González-Burgos G (2008). Neuroplasticity of neocortical circuits in schizophrenia. Neuropsychopharmacology.

[31] Martín A (2013). PET imaging of serotoninergic neurotransmission with [(11)C]DASB and [(18)F]altanserin after focal cerebral ischemia in rats. J. Cereb. Blood Flow Metab..

[32] De Lean A, Munson PJ, Rodbard D (1978). Simultaneous analysis of families of sigmoidal curves: application to bioassay, radioligand assay, and physiological dose-response curves. Am. J. Physiol..

[33] Motulsky MJ, Ransnas LA (1987). Fitting curves to data using nonlinear regression: a practical and nonmathematical review. FASEB J..

[34] Lal S, Nair NPV, Cecyre D, Quirion R (1993). Levomepromazine receptor binding profile in human brain - implications for treatment-resistant schizophrenia. Acta Psychiatr. Scand..

[35] Psychoactive Drug Screening Program (PDSP) database. https://pdsp.unc.edu/databases/kidb.php(2021).

[36] Shalgunov V (2019). Hunting for the high-affinity state of G-protein-coupled receptors with agonist tracers: theoretical and practical considerations for positron tomography imaging. Med. Res. Rev..

[37] Ettrup A (2016). Serotonin 2A receptor agonist binding in the human brain with [^11^C]Cimbi-36: test–retest reproducibility and head-to-head comparison with the antagonist [^18^F]altanserin. Neuroimage.

[38] McCullumsmith RE, Hammond JH, Shan D, Meador-Woodruff JH (2014). Postmortem brain: an underutilized substrate for studying severe mental illness. Neuropsychopharmacology.

[39] Jones H, Curtis VA, Wright P, Lucey JV (1998). Neuroendocrine evidence that clozapine’s serotonergic antagonism is relevant to its efficacy in treating hallucinations and other positive schizophrenic symptoms. Am. J. Psychiatry.

[40] Abel KM, O’Keane V, Murray RM (1996). Enhancement of the prolactin response to d-fenfluramine in drug-naive schizophrenia patients. Br. J. Psychiatry.

[41] Monteleone P, Tortorella A, Borriello R, Cassandro P, Maj M (1999). Prolactin hyperresponsiveness to D-fenfluramine in drug-free schizophrenia patients: a placebo controlled study. Biol. Psychiatry.

[42] Moreno JL (2016). Allosteric signaling through an mGlu2 and 5-HT2A heteromeric receptor complex and its potential contribution to schizophrenia. Sci. Signal..

[43] Brea J (2009). Evidence for distinct antagonist-revealed functional states of 5-hydroxytryptamine_2A_ receptor homodimers. Mol. Pharmacol..

[44] Gross-Isseroff R, Salama D, Israeli M, Biegon A (1990). Autoradiographic analysis of age-dependent changes in serotonin 5-HT2 receptors of the human brain postmortem. Brain Res..

[45] Karrer TM, McLaughlin CL, Guaglianone CP (2019). Reduced serotonin receptors and transporters in normal aging adults: a meta-analysis of PET and SPECT imaging studies. Neurobiol. Aging.

[46] Raval NR (2021). Single dose of psilocybin increases synaptic density and decreases 5-HT_2A_ receptor density in the pig brain. Int. J. Mol. Sci..

[47] Underwood MD (2018). Serotonin receptors and suicide, major depression, alcohol use disorder and reported early life adversity. Transl. Psychiatry.

[48] Muguruza C (2014). Evaluation of 5-HT_2A_ and mGlu_2/3_ receptors in postmortem prefrontal cortex of subjects with major depressive disorder: effect of antidepressant treatment. Neuropharmacology.

[49] Zhao J (2015). Different stress-related gene expression in depression and suicide. J. Psychiatr. Res..

