# Supplementary material for: Opposite alterations of 5­HT2A receptor brain density in subjects with schizophrenia: relevance of radiotracers pharmacological profile
Source: Transl Psychiatry. 2021 May 20;11:302. doi: 10.1038/s41398-021-01430-7 (PMC8137947; doi:10.1038/s41398-021-01430-7)
Supplement: Supplementary file 1 — Table S1 [file 41398_2021_1430_MOESM1_ESM.docx]

**Table S1** ***In vivo* PET neuroimaging and *post-mortem* radioligand binding studies of cortical 5-HT_2A_Rs in schizophrenia.**

| **Study** | **Type of study** | **Radioligand** | **Tissue preparation** | **Cohort size (n)** | |  | **Sex (M/F)** | | **Diagnosis method** | **APs in Sch** | **BA or cortical region** | **5-HT_2A_R binding sites** |
| --- | --- | --- | --- | --- | --- | --- | --- | --- | --- | --- | --- | --- |
|  |  |  |  | **Sch** | **C** |  | **Sch** | **C** |  |  |  |  |
| Bennett *et al.* 1979 ^50^ | *Postmortem* | [^3^H]LSD | Total homogenate | 21 | 31 |  | 12M/9F | 28M/3F | DSM-II (AM) | 12T/9F | 6,8-11,44-47 | ↓ All Sch |
| Whitaker *et al.* 1981 ^23^ | *Postmortem* | [^3^H]LSD | Total homogenate | 13 | 8 |  | 8M/5F | 4M/4F | Schneiderian Feighner (PM) | 8T/5F | 4,10,11 | ↑ Sch-F  NC Sch-T |
| Reynolds *et al.* 1983 ^51^ | *Postmortem* | [^3^H]Ketanserin | Total homogenate | 11 | 10 |  | - | - | - | - | 10 | NC All Sch |
| Mita *et al*.  1986 ^52^ | *Postmortem* | [^3^H]Ketanserin | Total homogenate | 11 | 9 |  | 7M/4F | 4M/5F | DSM-III (AM) | 7T/4F | 9 | ↓ Sch-F  ↓ Sch-T |
| Arora & Meltzer 1991 ^53^ | *Postmortem* | [^3^H]Spiperone |  | 11 | 11 |  | 10M/1F | 6M/5F | - | 11T | 8,9 | ↓ Sch-T |
| Joyce *et al.* 1993 ^54^ | *Postmortem* | [^125^I]LSD and  [^3^H]Ketanserin | Sections/  Autoradiography | 10 | 8 |  | 7M/3F | 4M/4F | DSM-III-R (AM) | 3T/7F | (A):6,24,9,4,23  (B):1,5,20-22,37,41,42 | NC All Sch (A)  ↑ All Sch (B) |
| Laruelle *et al.* 1993 ^55^ | *Postmortem* | [^3^H]Ketanserin | Total homogenate | (A)6  (B)10 | (A)13  (B)13 |  | (A)4M/2F  (B)7M/3F | (A)11M/2F  (B)9M/4F | DSM-III-R (AM) | (A)4T/2F  (B)6T/4F | (A):10  (B): 17,18 | ↓ Sch-F and Sch-T (A)  NC Sch-F and Sch-T (B) |
| Dean *et al.* 1996 ^56^ | *Postmortem* | [^3^H]Ketanserin | Membranes | 20 | 20 |  | 16M/4F | 16M/4F | DSM-III-R (PM) | - | 9 | NC All Sch |
| Dean & Hayes 1996 ^57^ | *Postmortem* | [^3^H]Ketanserin | Sections/  Autoradiography | 20 | 20 |  | 16M/4F | 17M/3F | DSM-III-R (PM) | 18T/1F/1- | 8,9,10 | ↓ All Sch |
| Burnet *et al.* 1996 ^58^ | *Postmortem* | [^3^H]Ketanserin | Sections/  Autoradiography | 13 | 15 |  | 8M/5F | 9M/6F | - | 12T/1F | 46 | ↓ All Sch |
| Gurevich & Joyce 1997 ^59^ | *Postmortem* | [^125^I]LSD | Sections/  Autoradiography | 10 | 13 |  | 6M/4F | 9M/4F | DSM-III-R (AM) | 4T/5F/1- | (A): 6,24  (B): 1-4,8,9,23, 31,32,40,44-46 | ↓ Sch-F and Sch-T (A)  NC Sch-F (B)  ↓ Sch-T (B) |

**Table S1 (Cont.) *In vivo* PET neuroimaging and *post-mortem* radioligand binding studies of cortical 5-HT_2A_Rs in schizophrenia.**

| **Study** | **Type of study** | **Radioligand** | **Tissue preparation** | **Cohort size (n)** | |  | **Sex (M/F)** | | **Diagnosis method** | **APs in Sch** | **BA or cortical region** | **5-HT_2A_R Binding sites** |
| --- | --- | --- | --- | --- | --- | --- | --- | --- | --- | --- | --- | --- |
|  |  |  |  | **Sch** | **C** |  | **Sch** | **C** |  |  |  |  |
| Trichard *et al.* 1998 ^8^ | PET | [^18^F]Setoperone |  | 14 | 15 |  | 10M/4F | 7M/8F | DSM-III-R | 7N/7F | Frontal, Parietal,  Temporal, Occipital | NC All Sch |
| Dean *et al.* 1998 ^60^ | *Postmortem* | [^3^H]Ketanserin | Sections/  Autoradiography | 55 | 55 |  | 41M/14F | 43M/12F | DSM-III-R (AM) | 55T | 9 | ↓ All Sch |
| Dean *et al.* 1999 ^61^ | *Postmortem* | [^3^H]Ketanserin | Sections/  Autoradiography | 19 | 19 |  | 15M/4F | 15M/4F | DSM-III-R (AM) | 17T/2F | 9 | ↓ All Sch |
| Lewis *et al.* 1999 ^62^ | PET | [^18^F]Setoperone |  | 13 | 26 |  | 10M/3F | 11M/15F | DSM-IV | 10N/3F | Prefrontal, Temporal, Parietal, Occipital | NC All Sch |
| Ngan *et al.* 2000 ^9^ | PET | [^18^F]Setoperone |  | 6 | 7 |  | 5M/1F | 4M/3F | DSM-IV | 6N | Frontal (VBA) | ↓ Sch-N |
| Verhoeff *et al.* 2000 ^10^ | PET | [^18^F]Setoperone |  | 13 | 35 |  | 11M/2F | 15M/20F | DSM-IV | 10N/3F | Neocortex (VBA) | NC All Sch |
| Okubo *et al*. 2000 ^11^ | PET | [^11^C]N-methylspiperone |  | 17 | 12 |  | 17M/0F | 12M/0F | DSM-III-R | 10N/7F | Prefrontal, Anterior cingulate, Temporal, Occipital | NC Sch-F  NC Sch-N |
| Pralong *et al.* 2000 ^63^ | *Postmortem* | [^3^H]Ketanserin | Sections/  Autoradiography  and Membranes | 20 | 20 |  | 17M/3F | 17M/3F | DSM-IV (AM) | 17T/3F | 22 | ↓ All Sch |
| Marazziti *et al.* 2003 ^64^ | *Postmortem* | [^3^H]Ketanserin | Membranes | 15 | 15 |  | 9M/6F | 9M/6F | DSM-IV (AM) | - | Frontal, Parietal | ↑ All Sch |
| Matsumoto *et al.* 2005 ^65^ | *Postmortem* | [^3^H]Ketanserin | Sections/  Autoradiography | 6 | 6 |  | 4M/2F | 4M/2F | DSM-III-R (AM) | 5T/1F | 9 | ↓ All Sch |

**Table S1 (Cont.) *In vivo* PET neuroimaging and *post-mortem* radioligand binding studies of cortical 5-HT_2A_Rs in schizophrenia.**

| **Study** | **Type of study** | **Radioligand** | **Tissue**  **preparation** | **Cohort size (n)** | |  | **Sex (M/F)** | | **Diagnosis method** | **APs in Sch** | **BA or cortical**  **region** | **5-HT_2A_R Binding sites** |
| --- | --- | --- | --- | --- | --- | --- | --- | --- | --- | --- | --- | --- |
|  |  |  |  | **Sch** | **C** |  | **Sch** | **C** |  |  |  |  |
| Hurlemann *et al.* 2005 ^66^ | PET | [^18^F]Altanserin |  | 6 | 7 |  | 5M/1F | 5M/2F | ERS | 6N | Orbitofrontal, Prefrontal, Cingulate, Occipital | ↓ Sch-N (OFC, PFC) |
| Hurlemann *et al.* 2008 ^22^ | PET | [^18^F]Altanserin |  | 14 | 21 |  | 10M/4F | 13M/8F | ERS | 14N | Neocortex, Sub- and Archicortex | ↓ Sch-N |
| Erritzoe *et al.* 2008 ^7^ | PET | [^18^F]Altanserin |  | 15 | 15 |  | 11M/4F | 11M/4F | ICD-10 and DSM-IV | 15N | Frontal, Parietal, Cingulate, Temporal, Sensory motor, Occipital | NC Sch-N |
| Dean et al. 2008 ^14^ | *Postmortem* | [^3^H]Ketanserin | Sections/Autoradiography (S)  Total homogenate (TH)  Membranes (M)  Cytosol (CT) | 14 | 14 |  | 9M/5F | 8M/6F | DSM-IV (PM) | 11T/3F | 9 | ↓ All Sch (S and TH)  NC All Sch (M and CT) |
| González-Maeso *et al.* 2008 ^24^ | *Postmortem* | [^3^H]Ketanserin | Membranes | 25 | 25 |  | 20M/5F | 20M/5F | DSM-IV (AM) | 12T/13F | 9 | ↑ Sch-F  NC Sch-T |
| Kang *et al.* 2009 ^67^ | *Postmortem* | [^3^H]Ketanserin | Sections/  Autoradiography | 8 | 8 |  | 8M/0F | 8M/0F | DSM-IV (PM) | 6T/2F | 22 | ↓ All Sch |
| Rasmussen *et al.* 2010 ^5^ | PET | [^18^F]Altanserin |  | 30 | 30 |  | 23M/7F | 23M/7F | ICD-10 and DSM IV | 30N | Frontal, Parietal, Cingulate, Temporal, Sensory motor, Occipital | ↓ Sch-N |
| Muguruza *et al*. 2013 ^13^ | *Postmortem* | [^3^H]Ketanserin | Membranes | 45 | 45 |  | 36M/9F | 36M/9F | DSM-IV (AM) | 16T/29F | 9 | ↑ Sch-F  NC Sch-T |
| Rasmussen *et al.* 2016 ^6^ | PET | [^18^F]Altanserin |  | 4 | 4 |  | 3M/1F | 3M/1F | - | 3F/1N | Frontal | ↓ All Sch |
| Radhakrishnan *et al.* 2020 ^68^ | PET | [^11^C]GSK215083 |  | 9 | 9 |  | 9M/0F | 9M/0F | DSM-IV | 9T | Frontal (VBA) | ↓ Sch-T |

Abbreviations: AM (*antemortem*); AP (antipsychotic); BA (Brodmann’s area); C (controls); CT (cytosol); ERS (elevated risk for schizophrenia); F (antipsychotic-free); M (membranes); M/F (male/female); N (antipsychotic-naïve); NC (no change); OFC (orbitofrontal cortex); PET (Positron emission tomography); PFC (prefrontal cortex); PM (*postmortem*); S (sections); Sch (schizophrenia subjects); T (antipsychotic-treated); TH (total homogenates); VBA (voxel-based analysis); - (not determined or not stated).

**References**

50. Bennett, J. P. Jr *et al*. Neurotransmitter receptors in frontal cortex of schizophrenics. *Arch. Gen. Psychiatry* **36**, 927-934 (1979).

51. Reynolds, G.P., Rossor, M.N. & Iversen, L.L. Preliminary studies of human cortical 5-HT2 receptors and their involvement in schizophrenia and neuroleptic drug action. *J. Neural Transm. Suppl.* **18**, 273-277 (1983).

52. Mita, T. *et al*. Decreased serotonin S2 and increased dopamine D2 receptors in chronic schizophrenics. *Biol. Psychiatry* **21**, 1407-1414 (1986).

53. Arora, R.C. & Meltzer, H.Y. Serotonergic measures in the brains of suicide victims: 5-HT2 binding sites in the frontal cortex of suicide victims and control subjects. *Am. J. Psychiatry* **146**, 730-736 (1989).

54. Joyce, J.N. *et al*. Serotonin uptake sites and serotonin receptors are altered in the limbic system of schizophrenics. *Neuropsychopharmacology* **8**, 315-336 (1993).

55. Laruelle, M. *et al*. Selective abnormalities of prefrontal serotonergic receptors in schizophrenia. A postmortem study. *Arch. Gen. Psychiatry* **50**, 810-818 (1993).

56. Dean, B. *et al*. Serotonin2 receptors and the serotonin transporter in the schizophrenic brain. *Behav. Brain Res.* **73**, 169-175 (1996).

57. Dean, B. & Hayes, W. Decreased frontal cortical serotonin2A receptors in schizophrenia. *Schizophr. Res.* **21**, 133-139 (1996).

58. Burnet, P.W., Eastwood, S.L. & Harrison, P. J. 5-HT_1A_ and 5-HT_2A_ receptor mRNAs and binding site densities are differentially altered in schizophrenia. *Neuropsychopharmacology* **15**, 442-455 (1996).

59. Gurevich, E.V. & Joyce, J.N. Alterations in the cortical serotonergic system in schizophrenia: a postmortem study. *Biol. Psychiatry* **42**, 529-545 (1997).

60. Dean, B., Hayes, W., Hill, C. & Copolov, D. Decreased serotonin2A receptors in Brodmann's area 9 from schizophrenic subjects. A pathological or pharmacological phenomenon? *Mol. Chem. Neuropathol.* **34**, 133-145 (1998).

61. Dean, B. *et al*. Changes in serotonin2A and GABA(A) receptors in schizophrenia: studies on the human dorsolateral prefrontal cortex. *J. Neurochem.* **72**, 1593-1599 (1999).

62. Lewis, R. *et al*. Serotonin 5-HT2 receptors in schizophrenia: a PET study using [^18^F]setoperone in neuroleptic-naive patients and normal subjects. *Am. J. Psychiatry* **156**, 72-78 (1999).

63. Pralong, D., Tomaskovic-Crook, E., Opeskin, K., Copolov, D. & Dean, B. Serotonin(2A) receptors are reduced in the planum temporale from subjects with schizophrenia. *Schizophr. Res.* **44**, 35-45 (2000).

64. Marazziti, D. *et al*. [^3^H]-ketanserin binding sites in different psychiatric disorders. *Neurochem. Int.* **42**, 511-516 (2003).

65. Matsumoto, I., Inoue, Y., Iwazaki, T., Pavey, G. & Dean, B. 5-HT_2A_ and muscarinic receptors in schizophrenia: a postmortem study. *Neurosci. Lett.* **379**, 164-168 (2005).

66. Hurlemann, R. *et al*. Decreased prefrontal 5-HT_2A_ receptor binding in subjects at enhanced risk for schizophrenia. *Anat. Embryol. (Berl)* **210**, 519-523 (2005).

67. Kang, K., Huang, X F., Wang, Q. & Deng, C. Decreased density of serotonin 2A receptors in the superior temporal gyrus in schizophrenia - a postmortem study. *Prog. Neuropsychopharmacol. Biol. Psychiatry* **33**, 867-871 (2009).

68. Radhakrishnan, R. *et al*. In vivo 5-HT_6_ and 5-HT_2A_ receptor availability in antipsychotic treated schizophrenia patients vs. unmedicated healthy humans measured with [^11^C]GSK215083 PET. *Psychiatry. Res. Neuroimaging* **295**, 111007 (2020).
