# Supplementary material for: Opposite alterations of 5­HT2A receptor brain density in subjects with schizophrenia: relevance of radiotracers pharmacological profile
Source: Transl Psychiatry. 2021 May 20;11:302. doi: 10.1038/s41398-021-01430-7 (PMC8137947; doi:10.1038/s41398-021-01430-7)
Supplement: Supplementary file 2 — Table S2 [file 41398_2021_1430_MOESM2_ESM.docx]

| **Table S2 Demographic characteristics, *post-mortem* conditions, cause of death and toxicological analysis of individual cases of antipsychotic-free and antipsychotic-treated subjects with schizophrenia (S) and their respective controls (C)** | | | | | | | | |
| --- | --- | --- | --- | --- | --- | --- | --- | --- |
|  |  |  |  |  |  |  |  |  |
| **Case** | **Gender (M/F)** | **Age (years)** | **PMI (hours)** | **Storage (months)** | **Cause of death** | **Mechanism of death** | **Antipsychotics in blood** | **Additional drugs in blood** |
|  |  |  |  |  |  |  |  |  |
| **S 1 ^a,b^** | M | 41 | 41 | 260 | Suicide | Gun shot | negative | diazepam, ethanol 0.17 g/L |
| **C 1 ^d^** | M | 41 | 20 | 205 | Accident | Road accident |  |  |
| **S 2 ^a,b^** | M | 49 | 41 | 266 | Suicide | Hanging | negative |  |
| **C 2** | M | 51 | 46 | 223 | Accident | Road accident |  |  |
| **S 3 ^a,b^** | M | 24 | 45 | 240 | Suicide | Jump from a height | negative |  |
| **C 3** | M | 22 | 37 | 246 | Accident | Road accident |  | ethanol 0.92 g/L |
| **S 4 ^a,b^** | M | 23 | 43 | 229 | Suicide | Hanging | negative |  |
| **C 4** | M | 22 | 32 | 247 | Accident | Road accident |  | ethanol 1.04 g/L |
| **S 5 ^d^** | M | 62 | 28 | 212 | Suicide | Jump from a height | negative |  |
| **C 5** | M | 62 | 44 | 262 | Accident | Road accident |  |  |
| **S 6** | F | 51 | 15 | 210 | Natural | Cardiorespiratory failure | negative |  |
| **C 6** | F | 51 | 62 | 204 | Accident | Road accident |  |  |
| **S 7 ^a,b,c^** | M | 31 | 14 | 147 | Suicide | Jump from a height | negative | lorazepam |
| **C 7** | M | 32 | 25 | 150 | Natural | Cardiorespiratory failure |  | amphetamine, ethanol 0.61 g/L |
| **S 8 ^b,c^** | M | 48 | 20 | 144 | Suicide | Jump in front of a train | negative |  |
| **C 8** | M | 47 | 17 | 165 | Accident | Road accident |  | nordiazepam |
| **S 9** | M | 23 | 13 | 157 | Suicide | Jump from a height | negative |  |
| **C 9** | M | 23 | 4 | 113 | Accident | Electrocution |  | ethanol 1.51 g/L |
| **S 10 ^b,c^** | M | 27 | 24 | 125 | Suicide | Gun shot | negative |  |
| **C 10 ^b,c^** | M | 29 | 18 | 89 | Accident | Fall from a height |  |  |
| **C (n=10)** | **9M/1F** | **38 (14.3)** | **31 (17.1)** | **190 (59)** |  |  |  |  |
| **S (n=10)** | **9M/1F** | **38 (14.1)** | **28 (13)** | **199 (52)** |  |  |  |  |

| **Table S2 (Cont.) Demographic characteristics, *post-mortem* conditions, cause of death and toxicological analysis of individual cases of antipsychotic-free and antipsychotic-treated subjects with schizophrenia (S) and their respective controls (C)** | | | | | | | | |
| --- | --- | --- | --- | --- | --- | --- | --- | --- |
|  |  |  |  |  |  |  |  |  |
| **Case** | **Gender (M/F)** | **Age (years)** | **PMI (hours)** | **Storage (months)** | **Cause of death** | **Mechanism of death** | **Antipsychotics in blood** | **Additional drugs in blood** |
|  |  |  |  |  |  |  |  |  |
| **S 11 ^a,b^** | F | 30 | 17 | 164 | Suicide | Jump from a height | haloperidol |  |
| **C 11** | F | 30 | 18 | 78 | Accident | Road accident |  |  |
| **S 12 ^a,b,d^** | M | 57 | 19 | 164 | Suicide | Jump in front of a train | quetiapine |  |
| **C 12 ^a,b^** | M | 58 | 19 | 246 | Accident | Road accident |  | ethanol 0.99 g/L |
| **S 13 ^b,c^** | M | 44 | 6 | 153 | Natural | Cardiorespiratory failure | levomepromazine / clotiapine |  |
| **C 13 ^a^** | M | 42 | 9 | 201 | Accident | Road accident |  |  |
| **S 14 ^a,b,c^** | M | 30 | 18 | 152 | Suicide | Jump from a height | olanzapine |  |
| **C 14 ^a,b,c^** | M | 30 | 12 | 152 | Accident | Road accident |  | tetrahydrocannabinol |
| **S 15 ^a,b,d^** | M | 56 | 8 | 151 | Suicide | Jump from a height | quetiapine |  |
| **C 15 ^d^** | M | 55 | 6 | 197 | Accident | Road accident |  |  |
| **S 16 ^a,b,d^** | M | 37 | 11 | 147 | Suicide | Jump from a height | olanzapine |  |
| **C 16** | M | 37 | 54 | 258 | Accident | Road accident |  | ethanol 2.2 g/L |
| **S 17 ^b,c^** | M | 23 | 16 | 142 | Suicide | Jump from a height | sulpiride |  |
| **C 17** | M | 22 | 25 | 198 | Accident | Road accident |  |  |
| **S 18 ^a,b,c^** | F | 35 | 3 | 142 | Suicide | Jump from a height | quetiapine |  |
| **C 18** | F | 35 | 17 | 210 | Accident | Road accident |  |  |
| **S 19 ^a,b,d^** | F | 56 | 13 | 141 | Natural | Cardiorespiratory failure | clozapine |  |
| **C 19** | F | 57 | 14 | 117 | Natural | Cardiorespiratory failure |  |  |
| **S 20** | M | 26 | 39 | 99 | Suicide | Jump from a height | olanzapine |  |
| **C 20** | M | 25 | 18 | 43 | Accident | Road accident |  |  |
| **C (n=10)** | **7M/3F** | **39 (13.4)** | **19 (13.4)** | **170 (71)** |  |  |  |  |
| **S (n=10)** | **7M/3F** | **39 (13)** | **15 (10)** | **146 (18)** |  |  |  |  |
| Values are mean (SD). F: female, M: male, PMI: post-mortem interval | | | | | | | | |

Some of the samples of the schizophrenia cases and controls have been previously used in evaluation of [^3^H]ketanserin binding sites [a: Gonzalez-Maeso *et al.* *Nature* **452**, 93-97 (2008); b: Muguruza *et al*. *Eur. Neuropsychopharmacology* **23**, 852-864 (2013)] and functional coupling of 5-HT_2A_R to G-proteins [c: García-Bea *et al*. *Eur. Neuropsychopharmacology* **29**, 1453-1463 (2019); d: Odagaki *et al.* *World J. Biol. Psychiatry* 9, in press].
